# A Rare Case of Left Main Coronary Artery Aneurysm

**DOI:** 10.7759/cureus.4190

**Published:** 2019-03-06

**Authors:** Iva N Dimitrova, Georgi Kotov, Alexandar Iliev

**Affiliations:** 1 Cardiology, St. Ekaterina University Hospital, Medical University of Sofia, Sofia, BGR; 2 Anatomy, Histology and Embryology, Medical University of Sofia, Sofia, BGR

**Keywords:** coronary artery aneurysm, left main coronary artery

## Abstract

Coronary artery aneurysms are uncommon and are usually described as dilatations larger than 1.5 times the diameter of the adjacent coronary arteries. The aneurysms vary between 0.15% and 4.9% and usually affect the right coronary artery, followed by the circumflex and anterior descending artery. Left main coronary artery (LMCA) aneurysm is an extremely rare clinical entity. Herein, we present a case in a 69-year-old man with a prior history of chest pain and palpitations. Significant ischemic ST changes were registered on Holter electrocardiography during paroxysmal atrial fibrillation.

## Introduction

Aneurysms of the coronary arteries are rarely reported in the literature. Their incidence varies between 0.15% and 4.9% of patients and they usually affect the right coronary artery, followed by the circumflex and anterior descending artery [1-2]. Left main coronary artery (LMCA) aneurysms are extremely rare and account for approximately 0.1% of coronary artery aneurysms [3]. Commonly, aneurysms can be divided into saccular or fusiform, single or multiple. Saccular aneurysms are usually spherical with larger transverse diameter, while fusiform ones have a larger longitudinal diameter. The term ‘aneurysm’ of the coronary artery is used when the dilatation exceeds the diameter of adjacent normal segments or the diameter of the patient's largest coronary vessel by 1.5 times [4]. Although rare, possible complications of coronary aneurysms have been reported. An aneurysm could become spastic, undergo thrombosis and spontaneous dissection and could lead to acute myocardial infarction [3,5].

Herein, we describe a case of a 69-year-old male presenting with chest oppression and palpitation, whose coronary angiogram revealed the presence of an LMCA aneurysm.

## Case presentation

A 69-year-old male of Europid origin with risk factors including high blood pressure, dyslipidemia, and diabetes presented to the Clinic of Cardiology at University Hospital St. Ekaterina with symptoms of chest oppression and palpitations. Holter electrocardiography monitoring registered paroxysmal atrial fibrillation with ischemic ST changes. An echocardiography scan showed preserved left ventricular ejection fraction (62%), low-grade mitral regurgitation, moderate aortic regurgitation and initial dilatation of the ascending aorta.

**Figure 1:**
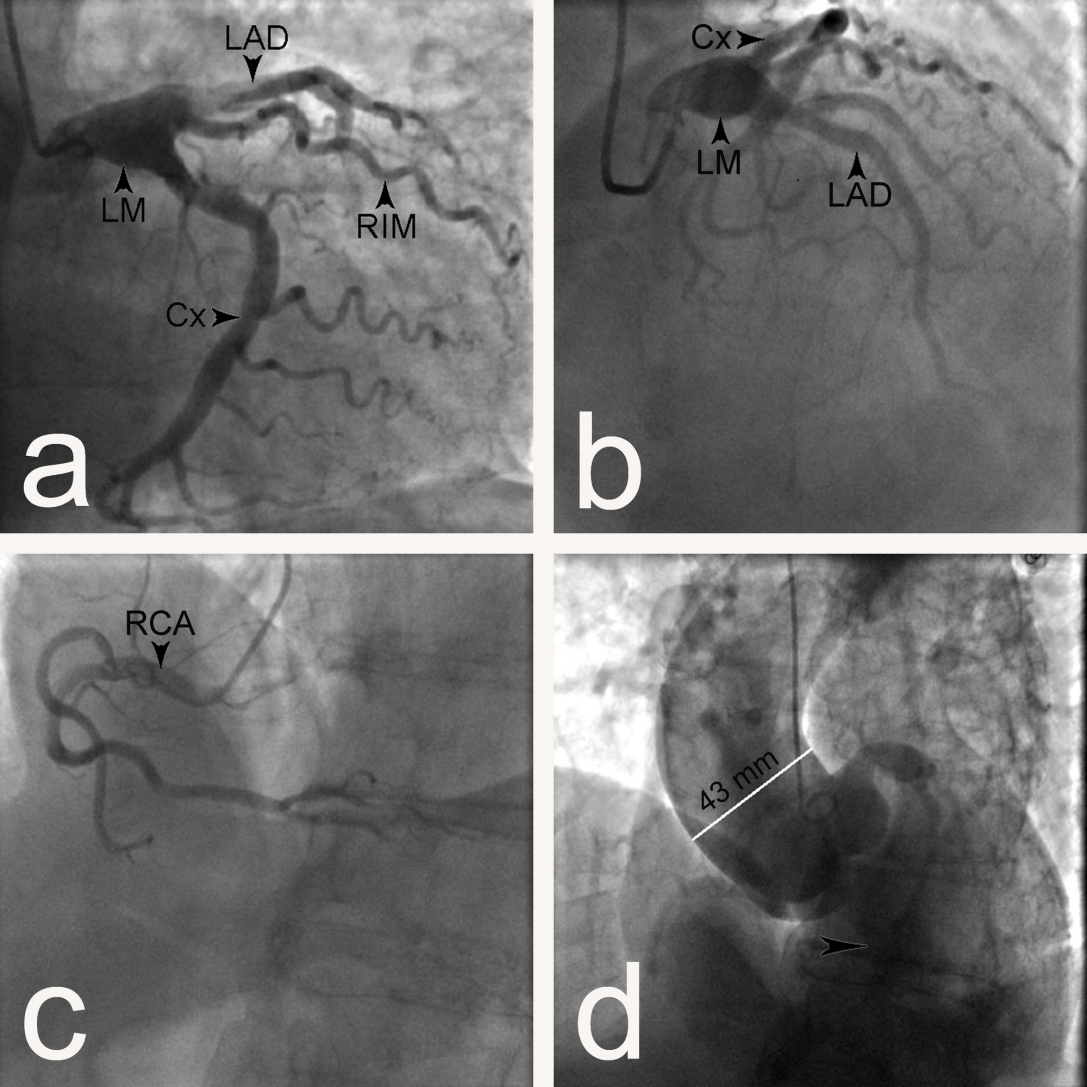
Coronary angiogram showing aneurysms of the left main coronary artery and the ascending aorta in a 69-year-old patient a, b) Left main coronary artery (LM) trifurcating into the left anterior descending artery (LAD), left circumflex artery (Cx), and ramus intermedius (RIM), a dilatation of the LM is visible; c) Right coronary artery (RCA) without any abnormalities; d) Dilatation of the ascending aorta with a transverse diameter of 43 mm. Arrowhead – aortic regurgitation.

The patient was planned for invasive diagnostic procedure. The coronary angiogram revealed an aneurysmal dilatation of the trunk of the LMCA with a transverse diameter of 11 mm (Figures 1A-1D).

We did not observe stenosis of the coronary arteries due to atherosclerosis. The conducted aortography revealed second-grade aortic regurgitation. The ascending aorta was dilated, with a transverse diameter of 43 mm. The patient was managed non-operatively with medication therapy including a vitamin K antagonist (acenocoumarol), antiarrhythmic (amiodarone), angiotensin II receptor blocker, β-blocker and lipid-lowering medication (statin).

## Discussion

Literature data on the treatment of LMCA aneurysms are scarcely presented. This could be due to their extremely rare incidence and unpredictable natural history. In a study of 22,000 coronary angiograms, Topaz et al. estimated its frequency to be only 0.1%. The largest width of this aneurysm was 1.9 cm [6]. Aneurysms have commonly been detected via coronary angiography or intravascular ultrasound and rarely in autopsy [2]. Angiography is the gold standard for diagnosis and treatment, but computed tomography (CT) and magnetic resonance (MR) coronary angiography may help increase detection [2].

The most common cause of coronary aneurysms is atherosclerosis, which accounts for half of the cases reported in adults [2]. Other reasons include congenital malformation, traumatic injury, previous balloon angioplasty, endocarditis, rheumatic fever, mycosis, syphilis, polyarteritis nodosa, systemic lupus erythematosus, Ehlers-Danlos syndrome, scleroderma, Marfan syndrome, Takayasu arteritis, Kawasaki disease, various genetic syndromes and idiopathic [5]. In our case, the aneurysm of the trunk of the LMCA coexisted with a dilatation of the ascending aorta. Thus, we discussed that these multiple aneurysms could be related to an underlying connective tissue disease.

Stabile et al. reported the formation of secondary aneurysms after using drug-eluting stents (DES) [7]. Aneurysmal formation is higher in DES than in bare metal stents (BMS) and may be associated with an increased risk for late stent thrombosis [8]. The incidence of aneurysms after DES implantation varies between 0.2% and 10.5% [9].

Coronary artery aneurysms are usually asymptomatic. However, the clinical manifestations may vary. A symptomatic coronary artery aneurysm may present with symptoms characteristic for coronary artery disease [10]. Pappy et al. reported that patients could present with symptoms of chest pain (angina pectoris), dyspnea, edema, myocardial infarction, and even sudden death. In our case, the patient presented with chest oppression and palpitations but significant coronary artery disease was not found on the subsequent coronary angiogram [11]. Thus, we discussed that the aneurysm of the LMCA described herein could have provoked the above symptoms.

The treatment of coronary artery aneurysms remains controversial. Different options include medical management, coil embolization, stent placement, and surgery. However, the choice of treatment option depends on the patient condition, concomitant diseases and infections, etiology, location, size and duration in time [2,12]. In our case, it was decided that the patient should be treated non-operatively and was managed with a medical therapy, including a vitamin K antagonist (acenocoumarol), antiarrhythmic (amiodarone), angiotensin II receptor blocker, β-blocker and lipid-lowering medication (statin).

## Conclusions

Coronary artery aneurysms are rare and commonly represent an accidental finding during coronary angiography. The proper therapy for these patients is not well established and certain controversies exists. In the present case, we reported a rare pathology, which was discussed to have been related to an underlying connective tissue disease, such as Kawasaki disease.
